# MicroRNA-dependent control of neuroplasticity in affective disorders

**DOI:** 10.1038/s41398-021-01379-7

**Published:** 2021-05-03

**Authors:** Helena Caria Martins, Gerhard Schratt

**Affiliations:** Lab of Systems Neuroscience, Institute for Neuroscience, Department of Health Science and Technology, Swiss Federal Institute of Technology ETH, 8057 Zurich, Switzerland

**Keywords:** Molecular neuroscience, Depression

## Abstract

Affective disorders are a group of neuropsychiatric disorders characterized by severe mood dysregulations accompanied by sleep, eating, cognitive, and attention disturbances, as well as recurring thoughts of suicide. Clinical studies consistently show that affective disorders are associated with reduced size of brain regions critical for mood and cognition, neuronal atrophy, and synaptic loss in these regions. However, the molecular mechanisms that mediate these changes and thereby increase the susceptibility to develop affective disorders remain poorly understood. MicroRNAs (miRNAs or miRs) are small regulatory RNAs that repress gene expression by binding to the 3ʹUTR of mRNAs. They have the ability to bind to hundreds of target mRNAs and to regulate entire gene networks and cellular pathways implicated in brain function and plasticity, many of them conserved in humans and other animals. In rodents, miRNAs regulate synaptic plasticity by controlling the morphology of dendrites and spines and the expression of neurotransmitter receptors. Furthermore, dysregulated miRNA expression is frequently observed in patients suffering from affective disorders. Together, multiple lines of evidence suggest a link between miRNA dysfunction and affective disorder pathology, providing a rationale to consider miRNAs as therapeutic tools or molecular biomarkers. This review aims to highlight the most recent and functionally relevant studies that contributed to a better understanding of miRNA function in the development and pathogenesis of affective disorders. We focused on in vivo functional studies, which demonstrate that miRNAs control higher brain functions, including mood and cognition, in rodents, and that their dysregulation causes disease-related behaviors.

## Introduction

Affective disorders are a group of neuropsychiatric diseases characterized by a complex set of symptoms that include disturbances in mood, sleep, cognition, attention, and recurring thoughts of suicide^1^. The two most prevalent disease presentations are major depressive disorder (MDD) characterized by severe and chronically lower mood and bipolar disorder (BD) with periods of depression, mania, hypomania, and mixed episodes. Both conditions are leading causes of disability^2,3^ and increased mortality^4,5^. Over the last 50 years, significant efforts were made into decoding the neurobiology of mood disorders. However, the pathological mechanisms are still largely unknown and the available pharmacotherapies are not fully efficacious^6^.

It is now accepted that the full manifestation of affective disorders requires an interaction between genetic predisposition (heritability estimates 37% for MDD^7^, higher than 60% for BD^8^) and environmental risk factors, such as childhood maltreatment or social isolation. Recent technical advances in the neuroscience field uncovered marked dysregulations of neuroplasticity in the brains of MDD patients. Particularly, neuronal atrophy, synaptic loss, and reduction in the volume of brain regions critical for mood regulation such as the hippocampus and medial prefrontal cortex (mPFC)^9–11^. The molecular mechanisms linking gene–environment interactions to defects in neuroplasticity, however, are still elusive.

MicroRNAs (miRNAs or miRs) are single-stranded, usually 19–22 nucleotide long RNAs, which belong to the class of small non-coding RNAs. They bind to the 3ʹUTR of one or multiple target transcripts and repress gene expression at the post-transcriptional level, either by promoting mRNA decay or by reducing mRNA translation levels^12^. Remarkably, miRNAs regulate up to 60% of all protein-coding genes^13^ and are often expressed in a brain region- or developmental stage-specific manner^14^. In this way, they control many biological processes within the developing and adult brain, which have been previously implicated in affective disorders. In rodents, miRNAs regulate the morphology of dendrites and spines, expression of neurotransmitter receptors, and synaptic plasticity^15^. In human studies, miRNA expression is often altered in MDD^16^ and BD^17^ patients. Altogether, miRNAs are very exciting targets for future therapeutic interventions or as prognostic and diagnostic biomarkers in affective disorders.

This review highlights the most relevant and recent advances in rodent model systems, which established miRNAs known to regulate neuroplasticity as potential mechanistic links between environmental stressors and gene expression changes that lead to the development of behavioral phenotypes of MDD and BD. Notably, miRNAs that modulate affective-like behavior control the expression of one or several genes primarily involved in signaling pathways activated by serotonin, glucocorticoids, neurotrophic factors, and Wnt (Table 1).

**Table 1:** List of miRNAs implicated in affective-like behavior through functional studies in animal models.

	Animal model	Behavioral phenotype	Molecular mechanism	Ref
Serotonin neurotransmission				
miR-16	KD of miR-16 in the rat hippocampus with an anti-miR-16.	Antidepressant-like effect in the FST and SPT.	Targets SERT and Bcl-2. Increases hippocampal neurogenesis.	^22^
	KD of miR-16 by i.c.v. injection of anti-miR-16 in rats.	Depressive-like phenotype in the FST and SPT.	Targets SERT. Decreased serotonin in the CSF.	^23^
miR-34 family	TKO mouse.	Antidepressant-like phenotype in the FST.	Prevents stress-induced release of serotonin in the mPFC.	^42^
	TKO mouse.	Antidepressant-like phenotype in the FST.	Targets CRFR1. Prevents stress-induced release of serotonin in the mPFC and GABA release in the BLA.	^43^
miR-135a	Overexpression of miR-135a in in serotonergic neurons of the raphe nuclei of mice.	Antidepressant-like phenotype in the FST.	Targets 5-HTR1A and SERT. Increases synaptic serotonin levels.	^44^
Glucocorticoid signaling				
miR-17–92 cluster	cKO mouse of miR-17–92 cluster in adult neural progenitors of the hippocampus.	Depressive-like phenotype in the FST, TST, and SPT.	Targets SGK1. Decreases the number of proliferative progenitors, and newborn neurons in the hippocampal DG.	^58^
miR-15	KD of miR-15a in BLA with a sponge lentivirus.	Anxiety-like phenotype in the elevated plus maze.	Regulated by glucocorticoids and targets FKBP51.	^59^
Neurotrophic factors				
miR-101	Overexpression of miR-101 in the VLO with a mimic	Antidepressant-like phenotype in the FST and SPT.	Targets DUSP1.	^67^
miR-182	Overexpression of miR-182 in the hippocampus with a lentivirus	Depressive-like phenotype in the FST, SPT, and NSFT.	Targets BDNF.	^73^
miR-323	KD of miR-323 in the mouse Cg1/2 with a sponge AAV.	Antidepressant-like phenotype in the TST.	Targets ErbB4.	^77^
Cytoskeletal-regulatory proteins				
miR-134	Overexpression in the rat mPFC with an AAV-miR-134.	Depressive-like phenotype in the FST and SPT.	Targets Limk1. Decreases dendritic spine density and synapse number in the vmPFC.	^79^
miR-212/132	tTA::miR-132 transgenic mice.	Moderate overexpression of miR-132 enhanced spatial memory and cognitive capacity in the Barnes maze task and NORT. Supra-physiological level of miR-132 impaired cognition.	Supra-physiological levels of miR-132 increase dendritic spine density in hippocampal neurons.	^102^
	AChE-R overexpressing TgR mice.	Cognitive impairments in the two-unit serial maze.	Targets AChE. Cholinergic hyper-excitation when exposed to pilocarpine.	^103^
	cKO of miR-132/-212 in excitatory neurons of the mouse forebrain.	Cognitive deficits in spatial memory, recognition memory, and in the NORT, Barnes maze, and contextual fear-conditioning tests.	miR-212 targets Stx1a. miR-132 targets Mash1.	^104^
	miR-132 transgenic and miR-132/212 knockout mice.	Anxiety-like phenotype in the elevated plus maze and open field assay.	Targets SIRT1 and PTEN.	^105^
miR-218	Overexpression in the mouse mPFC with an AAV-miR-218.	Antidepressant-like phenotype in the FST and SIT.	Targets DCC. Increases density of dendritic spines the mPFC.	^107^
Wnt signaling				
miR-124	KD of miR-124 in the rat hippocampus using lentivirus.	Antidepressant-like phenotype in the FST and SPT.	Targets BDNF.	^118^
	KD of miR-124 in the rat PFC using lentivirus.	Antidepressant-like phenotype in the FST, SPT, and NSFT.	Targets SIRT1.	^119^
	KD of miR-124 by i.c.v. injection of an antagomir in mice.	Antidepressant-like phenotype in the TST and SPT.	Targets GR. Increases hippocampal neuron proliferation.	^120^
	Overexpression of miR-124 in excitatory hippocampal neurons with an AAV-miR-124	Antidepressant-like phenotype in the SIT, SPT, and NSFT.	Targets HDAC4/5, and GSK3β. Increases spine density in the DG neurons.	^123^
	Overexpression of miR-124 in the mouse hippocampus with a lentivirus.	Antidepressant-like phenotype in the FST, TST, SPT, and SIT.	Targets STAT3. Decreases microglia activation.	^124^
miR-214	KD of miR-214 by i.c.v. injection of an antagomir in mice.	Antidepressant-like phenotype in the TST and SIT.	Targets β-catenin. Decreases amplitude of mEPSC, and number of dendritic spines in hippocampal neurons.	^125^
miR-221	KD of miR-221 by i.c.v. injection of an antagomir in mice.	Antidepressant-like phenotype in the FST, TST, and SPT.	Targets Wnt-2. Decreases neuronal proliferation and promotes neuronal apoptosis in the hippocampus.	^127^
New insights				
miR-9	Overexpression of CircDYM in the hippocampus. CircDYM acts as an endogenous sponge of miR-9.	Antidepressant-like phenotype in the FST and TST.	Targets HECTD1. CircDYM overexpression prevented miR-9-induced microglia activation.	^131^
miR-139	KD of miR-139 via intranasal administration of an antagomir in mice.	Antidepressant-like phenotype in the FST, TST, and NSFT.	Increases the number of immature neurons and newborn mature neurons in the mouse hippocampal DG.	^135^

*KD* knockdown, *5-HTR1A* 5-hydroxytryptamine/serotonin receptor 1A, *BDNF* brain-derived neurotrophic factor, *BLA* basolateral amygdala, *cKO* conditional knockout, *CRFR1* corticotropin-releasing factor receptor 1, *CSF* cerebrospinal fluid, *PTEN* phosphatase and tensin homolog, *DCC* deleted in colorectal cancer, *DG* dentate gyrus, *DUSP1* dual-specific phosphatase 1, *FKBP51* FK506-binding protein 51, *FST* forced swim test, *GABA* γ-aminobutyric acid, *GR* glucocorticoid receptor, *GSK3β* glycogen synthase kinase 3 beta, *HDAC4/5* histone deacetylase 4/5, *HECTD1* HECT domain E3 ubiquitin protein ligase 1, *i.c.v.* intracerebroventricular, *KD* knockdown, *Limk1* LIM motif-containing protein kinase 1, *miRNA or miR* microRNA, *mPFC* medial prefrontal cortex, *NORT* novel object recognition test, *NSFT* novelty-suppressed feeding test, *SERT* serotonin transporter, *SGK1* serum- and glucocorticoid-inducible protein kinase-1, *SIRT1* sirtuin 1, *SIT* social interaction test, *mEPSC* mini excitatory postsynaptic currents, *SPT* sucrose preference test, *STAT3* signal transducer and activator of transcription 3, *TKO* triple constitutive knockout, *TST* tail suspension test, *VLO* ventrolateral orbital cortex, *vmPFC* ventromedial prefrontal cortex.

### MicroRNAs that modulate serotonergic neurotransmission

Impaired neurotransmission mediated by serotonin (5-hydroxytryptamine/serotonin, 5-HT) is the most studied vulnerability factor of affective illnesses. Several lines of evidence suggest that a reduction in the 5-hydroxytryptamine/serotonin receptor 1A (5-HTR1A) binding causes the serotonin deficit observed in the cortex and hippocampus of MDD patients^18–20^. Consequently, the first treatment strategies focused on elevating the levels of monoamines in the brain. A classic example are selective serotonin reuptake inhibitors (SSRIs), like fluoxetine, which reduce presynaptic serotonin transporters’ (SERT) binding and protein levels, thereby preventing the reuptake of the neurotransmitter from the synaptic cleft and increasing its levels at the synapse^21^.

#### miR-16

**Fig. 1:**
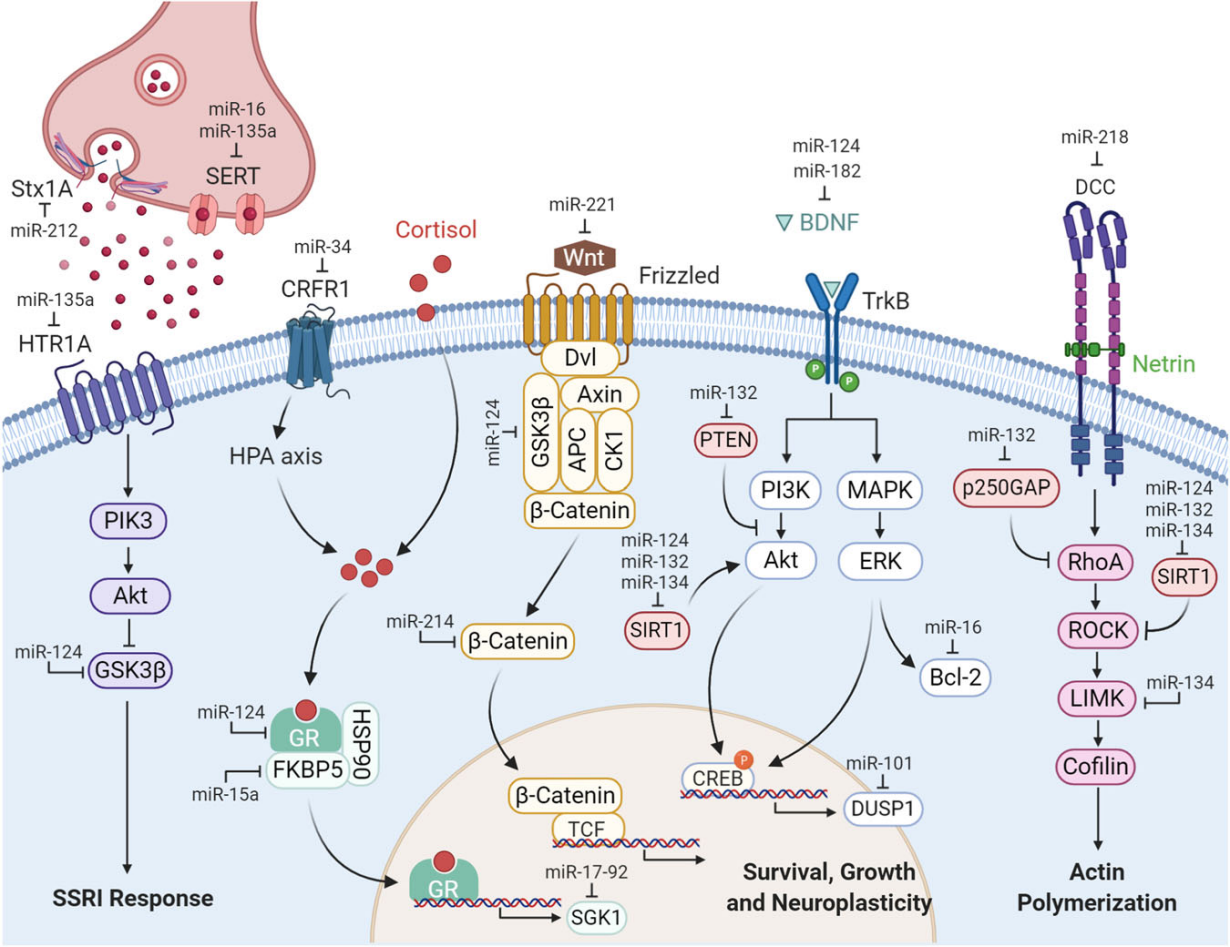
miRNAs affect animal behavior related to MDD and BD by regulating the expression of genes involved in serotonergic neurotransmission and signaling pathways activated by glucocorticoids, BDNF, Wnt, and guidance cues. Typical antidepressants, such as SSRIs, block monoamine reuptake by the SERT and, when chronically administered, lead to the desensitization of serotonin receptors (HTR)^21^. Signaling by glucocorticoids, BDNF, and Wnt controls multiple physiological functions such as neuronal survival, growth, and plasticity^61,110,149^. During development, the Netrin-1/DCC pathway plays a critical role in axonal outgrowth toward the prefrontal cortex and the organization of mPFC connectivity in adulthood^150^. Created with BioRender.com. Abbreviations: Stx1A, syntaxin 1A; SERT, serotonin transporter; HTR1A, serotonin receptor 1A; PIK3, phosphoinositide 3-kinase; Akt, protein kinase B; GSK3β, glycogen synthase kinase 3 beta; SSRI, selective serotonin reuptake inhibitor; CRFR1, corticotropin-releasing factor receptor 1; HPA axis, hypothalamus–pituitary–adrenal axis; GR, glucocorticoid receptor; FKBP5, FK506-binding protein 5; HSP90, heat shock protein 90; SGK1, serum- and glucocorticoid-inducible protein kinase-1; Wnt, Wingless-related integration site; Dvl, dishevelled protein; APC, adenomatous polyposis coli protein; CK1, casein kinase 1; TCF, T cell factor; BDNF, brain-derived neurotrophic factor; TrkB, neurotrophic receptor tyrosine kinase 2; PTEN phosphatase and tensin homolog; SIRT1, sirtuin 1; CREB, cAMP response element-binding protein; DUSP1, dual-specific phosphatase 1; MAPK, mitogen-activated protein kinase; ERK, extracellular signal-regulated kinase; Bcl-2, B-cell lymphoma 2; DCC, deleted in colorectal carcinoma; p250GAP, Rho GTPase activating protein 32; RhoA, Ras homolog family, member A; ROCK, Rho-associated coiled-coil kinases; LIMK, LIM motif-containing protein kinase.

The Kellermann lab published two very comprehensive studies demonstrating that fluoxetine interferes with SERT translation via miR-16 (Fig. 1). They showed that chronic injection of fluoxetine into the mouse serotonergic raphe nuclei increases serotonin by promoting the maturation of miR-16, which in turn decreases SERT expression by direct interaction with the SERT mRNA. Importantly, injection of miR-16 into the raphe and injection of anti-miR-16 into the locus coeruleus ameliorated the deterioration of the coat, reduction in body weight, sucrose preference, and locomotor activity induced by chronic stress to the same extent as fluoxetine^22^. Chronic antidepressant administration acted beyond the raphe by promoting the release of signaling molecules that antagonized the levels of miR-16 in the locus coeruleus and hippocampus^23^. Specifically in the hippocampus, reduced levels of miR-16 unlocked the translation of the anti-apoptotic and neurotrophic factor Bcl-2, which resulted in increased neurogenesis and facilitation of the antidepressant effect of fluoxetine^23^ (Fig. 1). Follow-up studies, however, were not able to fully recapitulate these initial findings. In humans, serum samples of MDD patients presented lower levels of miR-16, and subsequent neutralization of miR-16 by intracerebroventricular (i.c.v.) injection in rats produced depressive-like behaviors in the sucrose preference test (SPT) and forced swim test (FST)^24^. These discrepancies are found also in the expression studies where chronic stress was shown to both up-regulate^25–27^ and/or down-regulate^28,29^ miR-16. These inconsistencies are most likely the result of studying different stress models, at different time points during and after the stress, in different brain regions, and with different miRNA detection/normalization methods. Therefore, although the role of miR-16 as a readout for antidepressant treatment appears well established, it is still rather unclear whether miR-16 is causally involved in the pathophysiology of affective disorders.

#### miR-34 family

The miR-34 family (composed of miR-34a, b, and c) is one of the miRNAs for which differential expression is most consistently observed across genetic profiling, in vitro and in vivo studies of mood disorders. Genetic variation in miR-34b/c was significantly associated with higher susceptibility to MDD and correlated with negative life events and cognitive dysfunction^30,31^. Circulating levels of miR-34a and miR-34b/c were up-regulated in MDD patients when compared with healthy controls^32,33^, and higher expression levels of miR-34a were also measured in postmortem cerebellar tissue of BD patients^34^. In accordance with these results, the expression of miR-34a dropped significantly with the administration of antidepressants and mood stabilizers^35,36^. Its central role in the regulation of the stress response has been firmly established^37–41^, but direct evidence related to its functional role in affective disorders became available just recently. Lo Iacono et al. showed that chronic stress induces expression of miR-34a, particularly in the mouse raphe nuclei, which was required to develop a depressive-like response^42^. Notably, raphe nuclei conditional knockout (cKO) of the three miR-34s (TKO) reversed the increase in immobility induced by chronic stress in the FST^42^. Interestingly, Andolina et al. showed that the constitutive deletion of miR-34s also reduces the immobility of mice in the FST by preventing the activation of the raphe nuclei and hence the release of serotonin and γ-aminobutyric acid (GABA) in the mPFC and basolateral amygdala (BLA), respectively^43^. Similarly, Lo Iacono et al. observed that in the absence of miR-34, stressed mice lacked the induction of serotonin production in the mPFC normally present in wild-type (WT) mice^42^. Altogether, these findings support the role of miR-34 in modulating neurotransmission between brain regions important for cognitive and emotional responses. Furthermore, the prospect that by regulating miR-34 levels one can modulate an individual’s susceptibility to stress, makes miR-34 a strong downstream target for gene–environment interactions responsible for the development of affective disorders. Thus far, the function of miR-34 in the serotonergic raphe nuclei was linked to its target corticotropin-releasing factor receptor 1 (CRFR1)^43^ (Fig. 1). Nevertheless, it would be very interesting to investigate other possible targets more directly associated with neuronal transmission and plasticity, such as HTR2C or GRM7^39^. Another interesting finding was that although miR-34a is predominantly found in the raphe nuclei, miR-34c is highly expressed in the hippocampus^42^. Thus, it is very appealing to postulate that miR-34a, b, and c regulate different sets of target genes in a context-dependent manner, which in turn could be highly informative for the development of specific antidepressant therapies.

#### miR-135

miR-135 is a great example of the pleiotropic functions that can be exerted by miRNAs. Depending on the cell type, expression levels, and degree of miRNA activity, a miRNA will bind preferentially to a specific target producing different disease symptoms. Issler et al. demonstrated that in the raphe nuclei, miR-135a controls the expression of the serotonin autoreceptor HTR1A and transporter SERT, and in doing so, it mediates antidepressant response^44^ (Fig. 1). Although chronic social defeat stress (CSDS) did not alter the levels of miR-135a, acute and chronic SSRI antidepressant treatment robustly increased miR-135a expression levels in the raphe nuclei of both unstressed and stressed mice. In line with this observation, miR-135a overexpression specifically in serotonergic neurons did not change the baseline behavior of mice, however, it conferred resilience against chronic stress in tests of anxiety- and depressive-like behaviors. In the FST, after chronic stress, mice overexpressing miR135a spent significantly less time immobile than controls. On the other hand, miR-135a lentiviral knockdown (KD) in the raphe nuclei significantly attenuated the response to antidepressants resulting in higher immobility times after SSRI administration. Furthermore, mice overexpressing miR-135a had lower tissue concentration of serotonin and increased serotonin metabolism indicating that miR-135 decreases depressive symptoms by inducing the degradation of HTR1A and SERT, thereby increasing the synaptic levels of serotonin in the brain^44^. Intriguingly, Mannironi et al. showed that KD of miR-135 in the mouse amygdala led to increased spontaneous excitatory postsynaptic currents (EPSCs) and anxiety-like behavior. This effect was likely due to increased levels of the complexin-1 and complexin-2 target genes, both regulators of synaptic vesicle function^45^. In the Issler et al. study, the authors also measured lower levels of miR-135a in the blood of depressed patients^44^. Whilst the work on miR-135a paved the way for a better understanding of the serotonergic regulation in psychopathologies and advanced miR-135a as a potential biomarker for depression, it also raised new questions about potential off-target effects of miRNA manipulation. Manipulating the expression and/or activity of a given miRNA may be beneficial in one brain region but detrimental in a different region or tissue. Moreover, their ability to target multiple genes may also lead to the silencing of unintended targets not related to the disease. Other side effects related to the miRNA itself or the delivery method may include immune system activation, cellular toxicity, and saturation of the miRNA biogenesis machinery^46^. On the other hand, upon careful monitoring of unwanted side effects, chemical modifications and small changes to the miRNA sequence can help avoid off-target effects and optimize gene-silencing efficiency^47^.

### MicroRNAs that modulate glucocorticoid signaling

Chronic stress is a well-known risk factor for the development of affective disorders. Depressed patients consistently show hyperactivity of the hypothalamus–pituitary–adrenal (HPA) axis and increased levels of the glucocorticoid hormones, which are thought to be a consequence of impaired glucocorticoid receptor (GR) function. Additionally, antidepressant treatment ameliorates depressive symptoms by normalizing the HPA axis hyperactivity^48,49^. Therefore, miRNAs that target components of the GR signaling pathway are candidates to modulate the activity of the HPA axis and antidepressant action.

#### miR-17–92 cluster

The polycistronic miR-17–92 cluster is located on human chromosome 13 and codes for six miRNAs (miR-17, miR-18a, miR-19a, miR-20a, miR-19b-1, and miR-92a) with well-established roles as oncogenes^50^. Individual members of this cluster and its two mammalian paralogs (miR-106b~25 and miR-106a~363 clusters) were more recently associated with psychiatric disorders since they were shown to regulate heart^51,52^ and immune^53^ function, both of which are often compromised in mental illness^54,55^. In BD specifically, dysregulation of miR-106a and miR-106b has been consistently reported in profiling studies performed in blood^56^ and postmortem PFC^17,57^ of manic patients. The most relevant study concerning the causal role of miR-17–92 in behaviors related to affective disorders was published by Jin et al.^58^. They showed that a cKO of the miR-17–92 cluster in adult neural progenitor cells of the dentate gyrus (DG) decreased the number of proliferative progenitors and newborn neurons, possibly by regulating serum- and glucocorticoid-inducible protein kinase-1 (SGK1) (Fig. 1). miR-17–92 cKO mice spent significantly more time immobile in the FST and tail suspension test (TST) and consumed less sucrose in the SPT than control mice, all of which indicate enhanced depressive-like behavior in the absence of miR-17–92. Remarkably, overexpression of miR-17–92 had the opposite effect on both neurogenesis and depressive-like behaviors^58^. Taken together, this study showed in a very in-depth and complete manner that down-regulation of the miR-17–92 cluster upon chronic stress elicited a depressive phenotype in mice by impairing hippocampal neurogenesis. Thus, increasing the levels of miR-17–92 cluster miRNAs could represent a novel promising antidepressant strategy.

#### miR-15

Volk et al. showed that KD of miR-15a in the mouse BLA produces strong anxiolytic effects under CSDS^59^. After observing that miR-15a levels were elevated in the amygdala of stressed mice, the authors searched for behavioral changes upon overexpression of miR-15a. The elevation of miR-15a in the BLA was not sufficient to produce any changes. However, miR-15a KD animals showed a tendency to spend less time and travel less in the open arms of the elevated plus maze, which was significant following chronic social defeat. Volk et al. concluded that miR-15a is essential to form the appropriate behavioral response to chronic stress and its down-regulation specifically impairs the recovery process. Similarly, elevations in miR-15a were observed in the peripheral blood of subjects either treated with dexamethasone or experiencing childhood trauma. Interestingly, miR-15a targets FKBP51, a known inhibitor of the GR, which was previously implicated in affective disorders^60^ (Fig. 1). Therefore, it might be worth investigating whether enhanced FKBP51 expression is responsible for the anxiolytic effect of miR-15a KD. Besides, miR-15a was reduced in postmortem samples of PFC from BD patients, whereas miR-15b was up-regulated in the blood of individuals with high familial risk to develop BD. Taken together, these findings support the need for additional studies on miR-15 in the context of stress-induced psychiatric disorders.

### MicroRNAs that modulate neurotrophic factors

The brain-derived neurotrophic factor (BDNF), is a key regulator of neurogenesis and development, synaptic formation, maturation, and plasticity^61^ and was very early on connected with affective disorders. Observations that antidepressants induce the expression of BDNF in rodents and depressed patients led to the hypothesis that depression is associated with lower levels of BDNF and antidepressants act by restoring its levels^62^. Other neurotrophic factors, such as neuregulins (NRGs), bind to ErbB receptors and also promote neuronal development and plasticity^63,64^. Polymorphisms in the genes encoding NRG1 and ErbB4 were first associated with schizophrenia^65^, but since then accumulating evidence suggest that NRG1-ErbB4 signaling is associated with affective disorders, although the mechanism is still unkown^64^. Therefore, miRNAs that regulate BDNF and NRGs and/or downstream signaling genes are promising therapeutic targets.

#### miR-101

miR-101 dysregulation was first associated with mood disorders in the PFC of depressed individuals that committed suicide^16^. Later, it was shown that miR-101 is also down-regulated in the PFC of a rat model of MDD, the Flinders Sensitive Line while its target glutamate transporter SLC1A1 mRNA and protein levels were increased^66^. More recently, the functional relevance of miR-101 was assessed in vivo in the rat ventrolateral orbital cortex (VLO)^67^. Zhao et al. demonstrated that chronic unpredictable mild stress (CUMS) decreased the levels of miR-101 in the VLO in comparison with control animals while intra-VLO microinjection with a miR-101 mimic had an antidepressant effect. miR-101 overexpression in chronically stressed rats increased their sucrose preference in the SPT, to the same extent as fluoxetine, and decreased their immobility time in the FST. Chronic mild stress also increased the levels of dual-specific phosphatase 1 (DUSP1), a validated target of miR-101^68^ (Fig. 1), which has previously been implicated in the pathophysiology of MDD^69^. Stress-induced elevation of DUSP1 expression in turn resulted in lower levels of phosphorylated extracellular signal-regulated kinase (ERK) and BDNF. Importantly, miR-101 overexpression abolished the differential regulation of DUSP1, phospho-ERK, and BDNF expression between CUMS-exposed and control rats, suggesting that the ERK/BDNF pathway could be an important mediator of the antidepressant effect of miR-101 (Fig. 1). In future studies, exploring if miR-101 modulates ERK/BDNF-related processes like neuronal survival and synaptic plasticity^70,71^ in the context of affective disorders are warranted.

#### miR-182

The relationship between genetic variation in miRNA and susceptibility to depression was studied for the first time two decades ago^72^. The authors performed a mutational screening study and discovered a significant association between the T allele of the rs76481776 polymorphism in the pre-miR-182 and late insomnia in MDD patients. This likely represents a gain-of-function mutation since increased levels of mature miR-182 were produced from a pre-miR-182 carrying the rs76481776 compared to the WT allele. miR-182 overexpression in turn caused a significant down-regulation of the direct target gene CLOCK, which is involved in sleep/awake cycle regulation^72^. Consistent with these early findings, a more recent study showed that miR-182 depletion has an antidepressant effect in rats^73^. Li et al. observed an up-regulation of miR-182 and reduction of BDNF levels in the hippocampus of rats exposed to CUMS. Further overexpression of miR-182 in the hippocampus of stressed rats with a lentiviral injection significantly exacerbated the expression of depressive-like behaviors induced by CUMS, which was associated with a decrease in BDNF and CREB1 levels. Chronically stressed rats injected with the miR-182 lentivirus showed a significant increase in the latency to approach the food pellet in the novelty-suppressed feeding test (NSFT), lower sucrose preference in the SPT, and higher immobility time in the FST. Interestingly, both silencing of miR-182 and injection of BDNF in the hippocampus of stressed rats rescued the depressive-like phenotype in all tested behaviors. Behavioral rescue was accompanied by higher BDNF, CREB1, and pCREB1 protein levels. The authors further confirmed BDNF is a direct target of miR-182 and propose that miR-182-dependent inhibition of BDNF could be partially responsible for depression-associated phenotypes caused by stress^73^ (Fig. 1).

#### miR-323

Up-regulation of miR-323 levels was first reported in the frontal cortex of newborn rats after maternal stress^74^ and in the plasma of mild cognitive impairment patients^75,76^. However, very recently miR-323 was for the first time shown to be involved in depression^77^. Fiori et al. showed that miR-323a together with miR-204, miR-320b, and miR-331 was up-regulated in the dorsal anterior cingulate cortex (ACC) and the lateral habenula of individuals that committed suicide during an episode of MDD, compared to age-matched controls. miR-323 regulated the levels of its target ErbB4, which showed reduced levels in both the ACC and the habenula of depressed patients, and in HEK293T cells transfected with a miR-323 mimic. Behavioral phenotyping was carried out after bilateral injection in the mouse Cg1/2 region of AAVs bearing a miR-323 precursor or sponge to overexpress or KD miR-323, respectively. Mice with overexpression exhibited an anxiety-like phenotype in the open field and elevated plus maze test while inhibition of miR-323 resulted in less anxiety- and depressive-like behaviors, in particular higher mobility time in the TST. The levels of ErbB4 were also measured in the mouse ACC. KD mice showed significantly higher levels of ErbB4 while miR-323 overexpression resulted in a trend for decreased expression of Erbb4^77^. This data nicely correlates with a recent study by Wang et al.^78^. Here the authors showed that CSDS in mice decreased the protein levels of NRG1 and ErbB4 in the mPFC and hippocampus, and NRG1 lateral ventricle administration rescued the depression-like behavior of the CSDS mice^78^. Taken together, both studies implicate the NRG1-ErbB4 signaling pathway in the pathology of depression and the KD of miR-323 as a possible therapeutic target. Therefore, future studies should explore a possible neuronal function of miR-323 potentially underlying anxiety- and depression-like behaviors and whether miR-323 function is mediated by its target ErbB4.

### microRNAs that modulate cytoskeletal-regulatory proteins

Structural brain changes are a core manifestation of affective disorders. Brain-imaging techniques and postmortem studies consistently show a reduction in the volume, neuronal size, and loss of synapses in the mPFC and hippocampus of MDD patients^9^. Many of these changes are mimicked by chronic stress exposure in rodents^9^. The following miRNAs target cytoskeletal-regulatory proteins and are dysregulated upon stress, providing a potential mechanistic link between stress and affective disorder-associated neuromorphological changes.

#### miR-134

Fan et al. showed that CUMS significantly induced the expression of miR-134 in the rat ventromedial PFC (vmPFC) and that overexpression of miR-134 was sufficient to produce depressive-like behaviors^79^. Both chronic stress and injection of an AAV overexpressing miR-134 in the vmPFC of unstressed rats resulted in increased immobility times in the FST and anhedonia in the SPT. Abnormal behavior was accompanied by a reduction in dendritic spine density and synapse number. Infusion of an AAV-miR-134 sponge into the vmPFC blocked miR-134 function and significantly ameliorated changes in behavior and neuronal structure induced by CUMS. The authors went on to show that both chronic stress and miR-134 injection decreased the levels of the target gene LIM motif-containing protein kinase 1 (Limk1) and phosphorylation of the Limk1 substrate cofilin within the vmPFC^79^ (Fig. 1). Interestingly, Limk1 regulates actin dynamics by phosphorylating cofilin^80^ and Schratt et al. showed that miR-134 reduces dendritic spine size by inhibiting Limk1 translation in hippocampal neurons^81^. Thus, structural abnormalities induced by chronic stress in the brain could arise from the dysregulated expression of miRNAs that control actin dynamics. In further support of a “pro-depressive” role of miR-134, Shen et al. recently reported that environmental enrichment (EE) reversed the CUMS-induced depressive-like behavior via a miR-134/SIRT1 pathway. EE reduced the levels of miR-134 and induced SIRT1 protein expression leading to increased dendritic spine density, dendrite arborization, and expression of synaptic proteins in the rat hippocampus^82^ (Fig. 1). Apparently at odds with this functional data, lower levels of miR-134 were measured in the plasma of MDD and bipolar mania patients compared to healthy individuals as well as in the plasma, hippocampus, PFC, and olfactory bulb of rats exposed to CUMS^79,83^. Although miRNAs can be robustly detected in peripheral fluids, alterations in levels of circulating miRNAs and those from tissues are often inconsistent, suggesting the existence of tissue-specific mechanisms that control miRNA abundance. Future work exploring the correlation between miR-134 expression in the plasma and brain of healthy individuals and patients should help to evaluate the potential of miR-134 as a therapeutic target and/or a novel disease biomarker in affective disorders.

#### miR-212/132

The miR-212/132 cluster contains four miRNAs (miR-212, miR-212*, miR-132, and miR-132*) which are derived from the same intronic primary transcript. In the brain, the functional contribution of miR-132 was thoroughly researched in comparison to the other members of the cluster. It promotes neurite outgrowth and dendritic spine formation in an activity-dependent manner, primarily by targeting p250GAP (Fig. 1)^84–88^, and it is involved in brain plasticity^89,90^. Since BDNF is a known upstream regulator of miR-212/132 expression^91^, the miR-212/132 cluster was very early on associated with affective disorders, especially depression. miR-132 is increased in the serum^92^ and peripheral blood of MDD patients^93–95^ as well as in rodent models of depression^93,96^. Additionally, treatment with SSRI antidepressant drugs induced both miR-212^97^ and miR-132^98–100^, whereas electroconvulsive stimulation only induced the expression of miR-212^101^. Direct evidence from behavioral experiments that miR-132 modulates depressive- or manic-like behaviors is unfortunately lacking. However, higher levels of miR-132 were positively correlated with deficits in visual memory^94^ and poorer cognitive performance in attention and executive function^95^ in depressed patients. Therefore, miR-132 could specifically affect learning- and memory-related aspects of affective disorders. Hansen et al. were the first to suggest that miR-132 is an important regulator of cognition within a critical range of expression^102^. Endogenous miR-132 expression was induced in the hippocampus of mice undergoing a Barnes maze spatial memory task by ~1.5-fold. Induction to this level of expression using transgenic mice in the presence of doxycycline enhanced memory capacity. However, in the absence of doxycycline, miR-132 was elevated >3-fold over control levels and produced severe cognitive effects^102^. Shaltiel et al. showed that acute stress-induced deficits in cognition were a result of chronically high levels of miR-132 levels in the mouse hippocampus and the resulting suppression of its target gene acetylcholinesterase^103^. On the other hand, the conditional deletion of miR-212−132 in excitatory forebrain neurons using the cre/lox system led to cognitive deficits in spatial memory, recognition memory, and novel object recognition^104^. All the above studies provide evidence that, within a critical window of expression, miR-132 supports cognitive capacity. Deviation from this physiological expression level in either direction, e.g., caused by stress, would be expected to disturb cognitive homeostasis. In this regard, Aten et al. showed recently that chronic stress-induced miR-132 and miR-212 expression in the amygdala and that both overexpression and KO of miR-132 produced anxiety-related behaviors^105^. It would be very interesting to test whether stress-induced miR-132 also produces depressive-like behaviors or related cognitive impairments.

#### miR-218

The role of miR-218 in the development of mood disorders and the brain, in general, was only very recently uncovered. Torres-Berrío and Lopez et al. first reported a strong reduction in miR-218 in the PFC of two separate and independent cohorts of depressed patients who committed suicide in comparison with healthy sudden death controls. These findings were mirrored in mice, where miR-218 levels were reduced in mice susceptible to CSDS compared to those that were resilient^106^. Torres-Berrío et al. further assessed the involvement of miR-218 in the development of stress vulnerability following CSDS in mice^107^. Here, Torres-Berrío used the single social defeat (SSD) paradigm to show that microinfusion of an antagomir against miR-218 in the mPFC and intranasal infusion of the antagomir promoted stress susceptibility of mice in the social interaction test (SIT). Importantly, both methods resulted in increased immobility duration in the FST, showing that miR-218 KD by itself was sufficient to induce depressive-like behaviors. On the contrary, overexpression of miR-218 in pyramidal neurons of the mPFC prevented the development of social avoidance and alterations in spine morphology following CSDS, without any effect in control mice. miR-218 overexpression prevented the stress-induced decrease in the density of dendritic spines in the mPFC of socially defeated mice, more specifically the reduction in the density of thin spines. Torres-Berrío and Lopez et al. established the Netrin-1 guidance cue receptor DCC (deleted in colorectal cancer) as a critical miR-218 target by showing that increased DCC expression in PFC neurons caused susceptibility to stress-induced depressive-like behaviors, whereas DCC deletion was protective (Fig. 1). Taken together, these studies provide rather compelling evidence that a reduction in miR-218 levels in the mPFC induces depressive-like behaviors in mice by rendering them more susceptible to chronic stress. This work raised many interesting questions, which were in part addressed in follow-up studies. Rocchi et al. showed recently that miR-218 overexpression up-regulates the GluA2 subunit and increases glutamatergic synaptic transmission at both single-neuron and network levels^108^, which is consistent with a previous observation that miR-218 rescued the stress-induced loss of dendritic spines^107^. Moreover, Manitt et al. showed that during adolescence DCC expression in dopaminergic neurons is required for appropriate organization of synaptic connectivity within the mPFC^109^. Therefore, dysregulations in miR-218 and DCC expression during adolescence, a critical period for stress-related disorders, may hinder mPFC wiring and glutamatergic neurotransmission in mood-related circuits that will increase the predisposition to develop affective disorders. Along these lines, it would be very interesting to explore whether changes in miR-218 in susceptible mice are already present before chronic stress or are rather a consequence of stress, and whether the protective effect of miR-218 against stress is indeed a result of enhanced synaptic connectivity, particularly related to the glutamatergic system. Torres-Berrío et al. also showed that mice susceptible to CSDS exhibit reduced levels of miR-218 in blood^107^. Thus, it is very appealing to pursue miR-218 as a biomarker and assess whether miR-218 is altered in the blood of MDD patients and possibly correlates with disease symptom severity and antidepressant treatment.

### MicroRNAs that modulate Wnt signaling

Wnt signaling is a highly conserved signal transduction pathway important for the survival, growth, and plasticity of neurons^110^. It was first linked to affective disorders by the observation that the BD drug lithium modulated the activity of two important components of the pathway, GSK3β and β-catenin^111^. Later, Wnt2 expression was shown to be induced by different classes of antidepressants and to produce antidepressant-like phenotypes when overexpressed in the rat hippocampus^112^. Below, we further discuss how specific miRNAs regulate the Wnt pathway at different levels to influence depressive-like behaviors.

#### miR-124

miR-124 is abundantly expressed in the brain where it drives neuronal differentiation and neurogenesis^113,114^. In rodents, miR-124 expression is induced by early-life stress^115–117^, chronic stress^118,119^, as well as in the hippocampus of mice chronically treated with corticosterone (CORT)^120^. Likewise, in MDD patients, miR-124 is up-regulated in both blood and postmortem PFC^100,121,122^. At the behavioral level, manipulation of miR-124 produced conflicting effects. On one hand, suppression of miR-124 using lentiviruses ameliorated the depressive-like phenotype of chronically stressed mice in the SPT and FST^118,119^. In addition, the administration of a miR-124 antagomir had beneficial effects on depression-related behaviors and partly reversed the deficits in hippocampal dendritic spine density and neuronal proliferation induced by CORT^116^. On the other hand, Higuchi et al. observed that chronic stress reduced the levels of miR-124, an effect that was blocked by antidepressant treatment^123^. Also, overexpression of miR-124 specifically in excitatory hippocampal neurons using AAV injection blocked the development of depressive-like behaviors induced by chronic stress in the SPT. In the same study, inhibition of miR-124 increased the behavioral susceptibility to mild repeated restraint stress (MRRS). Interestingly, Higuchi also observed that miR-124 modulates neuronal morphology under stress. However, contrary to the results of Wang et al.^120^, hippocampal inhibition of miR-124 enhanced the stress-induced loss of dendritic spines and decreased dendritic length of DG neurons, both of which were rescued by overexpression of miR-124. Moreover, pharmacological inhibition of the targets GSK3 and HDAC4/5 resulted in antidepressant-like behavioral effects^123^. Overall, this study suggests that down-regulation of miR-124 and the concomitant up-regulation of GSK3 and HDAC4/5 are causally involved in the atrophy of hippocampal neurons induced by chronic stress (Fig. 1). In further support of a protecting effect of miR-124, Lou et al. showed recently that restoring miR-124 levels in conditions of chronic stress alleviated the increased immobility in the TST and FST and inhibited microglial activation, possibly by degrading STAT3 mRNA^124^. Discrepancies within in vivo studies may arise from different sources. In the case of miR-124, different genetic backgrounds of the animal models, stress paradigms, cell type, and developmental stage, likely resulted in different behavioral outcomes. Further studies are required to clarify whether the primary function of miR-124 is “pro- or anti-depressant”.

#### miR-214

Until very recently the role of miR-214 in neuronal processes and the etiology of affective disorders was very much unknown. However, Deng et al. uncovered a novel function of miR-214 in synaptic plasticity and showed that miR-214 is involved in the pathophysiology of MDD induced by CSDS in mice by targeting the Wnt pathway component β-catenin both in vitro and in vivo. Two models of depression, CUMS and CSDS, elevated the expression of miR-214 and decreased β-catenin protein levels in the mPFC of depressed mice. KD of miR-214 by i.c.v. injection of an antagomir increased the levels of β-catenin and improved the depressive-like phenotype of socially defeated mice. Compared to defeated mice, miR-214 antagomir-treated animals spent more time socially interacting with an unfamiliar target in the SIT and spent less time immobile in the TST, without any significant effect in the SPT. Lentiviral overexpression of β-catenin produced the same results, showing that miR-214/β-catenin is important for depressive-like behavior expression but not anhedonia. Interestingly, the silencing of miR-214 prevented the decrease in amplitude of mEPSC and the number of dendritic spines in hippocampal neurons of depressed mice^125^. This result suggests that miR-214 antagomir acts as an antidepressant by elevating the levels of β-catenin in the mPFC and improving the maladaptive synaptic plasticity induced by chronic stress in mice (Fig. 1). Dias et al. also showed that overexpression of β-catenin in the mouse NAc had a pro-resilient, anxiolytic, and antidepressant-like effect^126^. Additionally, using β-catenin ChIP-seq and qPCR, the authors validated the miRNA biogenesis gene Dicer 1 as a direct transcriptional target of β-catenin^126^. The findings by Deng et al. and Dias et al. established a novel connection between Wnt signaling and miRNAs in the brain, whereby β-catenin promotes resilience by enhancing the biogenesis of specific miRNAs, such as miR-214^125,126^. Finally, Deng et al. pointed to a potential non-invasive method of therapeutically delivering the antagomir to the brain. Intranasal delivery of antagomir-214 produced the same antidepressant effect as i.c.v. injection in the SIT and TST, which was abolished by intracranial injection of a miR-214 agomir^125^. Therefore, the intranasal administration of short oligonucleotide targeting miRNAs might represent a particularly promising novel strategy for miRNA therapeutics in affective disorders.

#### miR-221

miR-221 has been consistently found elevated in plasma and cerebrospinal fluid (CSF) samples of MDD patients^32,127,128^. Furthermore, in vitro treatment of human cell lines with the SSRI paroxetine significantly decreased the levels of miR-221 while increasing the levels of its target integrin subunit beta 3 (ITGB3)^129,130^. Building upon these findings, Lian et al. showed that miR-221 is up-regulated in the hippocampus of mice subjected to CUMS and further characterized its relevance in vivo^128^. Intracerebroventricular injection of an antagomir to silence miR-221 prevented the decrease in sucrose preference in the SPT and decreased the immobility times in the FST and TST of stressed mice. It also reversed the decrease of its target Wnt2 as well as phospho-CREB and BDNF protein levels that were induced by CUMS. On the other hand, overexpression of miR-221 by injection of an agomir reversed the treatment of fluoxetine on CUMS mice in the SPT and FST behavioral tests, and the induction of Wnt2, phospho-CREB, and BDNF. Using primary mouse hippocampal neurons, the authors showed that miR-221 is a negative regulator of neuronal proliferation and promoted neuronal apoptosis by modulating the expression of Wnt2. Together, these findings suggest that chronic stress inhibits hippocampal neuronal proliferation and promotes apoptosis by down-regulating the Wnt2/CREB/BDNF axis via miR-221 (Fig. 1). Altogether, miR-221 might represent a promising candidate target for future therapeutic strategies.

### New insights

Despite the considerable efforts mentioned in the present review, the pursuit of better diagnostic and therapeutic strategies for affective disorders has yet to advance suitable targets. Therefore, we wanted to highlight recent studies that, due to their interest and novelty, break new ground for future research avenues in the field.

Zhang et al. published the first in vivo evidence of a circular RNA (circRNA)-miRNA-mRNA regulatory network important for the development of depressive-like behaviors^131^. CircDYM, which has one target site for miR-9, was down-regulated in the blood of MDD patients as well as in the plasma and hippocampus of mice exposed to chronic unpredictable stress (CUS). Concomitantly, miR-9 expression was significantly increased in both cases. Overexpression of CircDYM in the hippocampus prevented the CUS-induced depressive-like behaviors in the FST and TST. At the molecular level, the authors demonstrated that CircDYM, acting as a miR-9 sponge, led to an up-regulation of miR-9 target gene HECT domain E3 ubiquitin protein ligase 1 (HECTD1), ubiquitination of heat shock protein 90 (HSP90), and reduced microglia activation. The findings open a new avenue to study circRNAs as potential biomarkers or an alternative therapeutic strategy to regulate miRNA levels in affective disorders.

Another promising research field studies exosomal miRNAs as potential biomarkers for neuropsychiatric disorders^132,133^. In a very interesting and innovative study, Wei et al. showed that miR-139 is strongly up-regulated in blood-derived exosomes of 33 MDD patients^134^. In mice, intranasal administration of a miR-139 antagomir ameliorated depressive-like behaviors by preventing the CUMS-mediated increase in miR-139 expression. These behavioral effects of miR-139 inhibition were paralleled by a decrease in hippocampal neurogenesis. Interestingly, mice injected in the tail vein with exosomes isolated from the blood of MDD patients developed depressive-like behaviors and showed significantly reduced neurogenesis. On the contrary, exosomes derived from healthy individuals and peripherally injected into chronically stressed mice had an antidepressant effect^134^. In further support for the role of exosomal miRNAs in the pathophysiology of affective disorders, Amoah et al. uncovered an astrocyte-enriched and exosome-secreted miRNA significantly increased in the orbitofrontal cortex (OFC) of BD and schizophrenia patients with a positive history of psychosis at the time of death^135^. This up-regulation correlated with lower expression levels of its targets glutamate ionotropic receptor NMDA-type subunit 2B (GRIN2B) and glutamate ionotropic receptor AMPA-type subunit 2 (GRIA2). Antipsychotic treatment reduced miR-223 synthesis in neurons but increased miR-223 exosomal secretion in astrocytes. Notably, the addition of astrocytic exosomes containing miR-223 to cortical neuronal cultures resulted in increased neuronal miR-223 expression and reductions in neuronal Grin2b, which were reversed by inhibition of miR-223 in astrocytes. In this context, it would be very interesting to investigate if astrocytic miR-223, which is transferred to neurons via exosomes, could also regulate synaptic plasticity and behavior. Taken together, both studies show that exosome-enriched miRNAs are dysregulated in affective disorders and that their secretion can regulate gene expression in neurons and ultimately animal behavior. However, it should be considered that exosomes could target other molecules in the periphery that indirectly regulate gene expression in the brain, a concern also acknowledged by the authors. Future studies will need to characterize the full spectrum of mRNAs, proteins, and miRNAs present in exosomes and to demonstrate a causal relationship between altered exosomal miRNAs and the deregulation of their target genes in the brain.

## Conclusions

Major depression and bipolar disorder are leading causes of disability worldwide and represent a global burden for society. Genetic studies made significant advances in identifying genetic variants that increase the susceptibility to develop mood disorders. We now understand that mood disorders arise from a complex interaction of multiple genes with the environment. As a result, the traditional approach of manipulating a single gene in rodent models have yielded very little success simulating the clinical setting.

MicroRNAs are negative regulators of gene expression, with known biological functions critical for the development and physiology of mammals. Notably, each miRNA can regulate hundreds of targets, and often one mRNA contains many conserved sites for the same or different miRNAs^136^. In the brain, a subset of miRNAs localize at synapses and control the expression of genes locally during synapse development and plasticity^15^. Therefore, their potential to control higher brain functions through the regulation of entire gene networks puts them at the forefront of preclinical research for psychiatric disorders.

In this review, we highlighted the most promising miRNAs that regulate neuroplasticity and animal behavior related to affective disorders. Interestingly, miRNAs that modulate affective-like behavior regulate the expression of one or several genes primarily involved in signaling pathways activated by serotonin, glucocorticoids, neurotrophic factors, and Wnt. Moreover, they respond to stress, neuronal activity, and antidepressant treatments. Therefore, directly targeting these miRNAs has great potential for the treatment of affective disorders. Molecular tools such as antagomirs and “sponges” or miRNA mimics and precursors can be delivered to silence or overexpress miRNAs, respectively^137^. Additionally, the introduction of mutations and single nucleotide polymorphisms (SNPs) in genes encoding for miRNAs, miRNA promoters, seed sequences, or 3ʹUTRs of target genes via CRISPR/Cas9 or AAV/lentiviral approaches in vivo will be very useful to understand miRNA function and related molecular pathways. Combining these tools with viral vectors, light activation or brain region or cell-type-specific Cre-driver transgenic mouse lines should further increase spatial and temporal resolution^137,138^. Treatment with antidepressants and antipsychotics often shows undesired side effects, such as weight gain and metabolic changes^139,140^. In this regard, cell-type-specific manipulation of miRNA activity represents an advantage over the more conventional psychopharmacological therapies as it could spare structures important for other functions, such as appetite regulation.

Throughout this review, we also discussed some important challenges that we believe need to be met to successfully expand the field of miRNA-based therapeutics directly or as novel non-invasive molecular markers. The first one being the conflicting results between some of the genetic and functional studies. We believe they arise from the different genetic backgrounds of the animal models, different stress paradigms, brain regions, developmental stages, and cell types. Importantly, this divergence stems also from the fact that animal models cannot fully mirror the complex and heterogeneous set of symptoms of the human affective disorder and that most animal studies, until recently, were done only in males^141–144^. Men and women possess inherent differences in their response to stress, clinical presentation, and response to treatment which leads to an altered susceptibility to develop mood disorders^145,146^. As highlighted by Simchovitz-Gesher and Soreq, it is especially important to study male–female differences at the transcriptome level^147^. Recent studies have shown the existence of sex-specific transcriptional patterns in the brain associated with psychiatric disorders, including MDD and BD. These differences are thought to be driven by the sex chromosomes. Importantly, miRNAs can regulate the transcription of homologous genes on both X and Y chromosomes or on one of them alone revealing a possible mechanism for the sexual dimorphism observed in affective disorders^147^. To fully decode the significance of miRNAs in the future of diagnosis and therapeutics, we should combine molecular studies with high-throughput approaches to look for miRNA profiles in animal models of disease as well as patients with big enough sample sizes and statistical power that include both sexes, different degrees of disease severity, and responsiveness to antidepressants/mood stabilizers. This would help to screen for patient-specific susceptibilities and develop efficacious therapies. Furthermore, establishing strong links between genetic variation and function will be extremely useful when predicting the effect of a mutation or SNP on disease susceptibility and progression as well as the choice and effectiveness of a therapeutic intervention. Future studies showing whether such mutations or SNPs lead to gain- or loss-of-function effects and the downstream cellular changes are paramount to the development of genome-based therapeutics. A second challenge is the polygenic nature of affective disorders that is difficult to recapitulate in animal models. Using induced pluripotent stem cell-derived neurons isolated from patients and targeting miRNAs or miRNA genes via CRISPR engineering is a promising strategy to better understand how the different genetic inputs relay the risk and development of affective disorders^148^. Finally, non-invasive, efficient delivery of compounds such as miRNA mimics, precursors, antagomirs, or miRNA sponges to the brain is still challenging. However, exciting alternatives to surpass this challenge are explored in recent studies showing promising results with exosomes and intranasal delivery^125,134^.

## References

[1] American Psychiatric Association. *Diagnostic and Statistical Manual of Mental Disorders*, 5th edn (DSM-5) (APA, 2013).

[2] Papakostas GI (2004). Quality of life assessments in major depressive disorder: a review of the literature. Gen. Hosp. Psychiatry.

[3] Michalak EE, Yatham LN, Lam RW (2005). Quality of life in bipolar disorder: a review of the literature. Health Qual. Life Outcomes.

[4] Moller HJ (2003). Suicide, suicidality and suicide prevention in affective disorders. Acta Psychiatr. Scand..

[5] Cuijpers P, Smit F (2002). Excess mortality in depression: a meta-analysis of community studies. J. Affect. Disord..

[6] Warden D, Rush AJ, Trivedi MH, Fava M, Wisniewski SR (2007). The STAR*D project results: a comprehensive review of findings. Curr. Psychiatry Rep..

[7] McMahon FJ (2018). Population-based estimates of heritability shed new light on clinical features of major depression. Am. J. Psychiatry.

[8] Johansson V, Kuja-Halkola R, Cannon TD, Hultman CM, Hedman AM (2019). A population-based heritability estimate of bipolar disorder – in a Swedish twin sample. Psychiatry Res..

[9] Duman RS, Aghajanian GK (2012). Synaptic dysfunction in depression: potential therapeutic targets. Science.

[10] Abdallah CG, Sanacora G, Duman RS, Krystal JH (2015). Ketamine and rapid-acting antidepressants: a window into a new neurobiology for mood disorder therapeutics. Annu. Rev. Med..

[11] Duman RS, Aghajanian GK, Sanacora G, Krystal JH (2016). Synaptic plasticity and depression: new insights from stress and rapid-acting antidepressants. Nat. Med..

[12] Filipowicz W, Bhattacharyya SN, Sonenberg N (2008). Mechanisms of post-transcriptional regulation by microRNAs: are the answers in sight?. Nat. Rev. Genet..

[13] Friedman RC, Farh KK-H, Burge CB, Bartel DP (2009). Most mammalian mRNAs are conserved targets of microRNAs. Genome Res..

[14] Krichevsky AM (2003). A microRNA array reveals extensive regulation of microRNAs during brain development. RNA.

[15] Schratt G (2009). microRNAs at the synapse. Nat. Rev. Neurosci..

[16] Smalheiser NR (2012). MicroRNA expression is down-regulated and reorganized in prefrontal cortex of depressed suicide subjects. PLoS ONE.

[17] Moreau MP, Bruse SE, David-Rus R, Buyske S, Brzustowicz LM (2011). Altered microRNA expression profiles in postmortem brain samples from individuals with schizophrenia and bipolar disorder. Biol. Psychiatry.

[18] Bhagwagar Z, Rabiner EA, Sargent PA, Grasby PM, Cowen PJ (2004). Persistent reduction in brain serotonin1A receptor binding in recovered depressed men measured by positron emission tomography with [11C]WAY-100635. Mol. Psychiatry.

[19] Hirvonen J (2008). Decreased brain serotonin 5-HT1A receptor availability in medication-naive patients with major depressive disorder: an in-vivo imaging study using PET and [carbonyl-11C]WAY-100635. J. Neuropsychopharmacol..

[20] Savitz J, Lucki I, Drevets WC (2009). 5-HT1A receptor function in major depressive disorder. Prog. Neurobiol..

[21] Malhi GS, Mann JJ (2018). Depression. Lancet.

[22] Baudry A, Mouillet-Richard S, Schneider B, Launay J-M, Kellermann O (2010). iR-16 targets the serotonin transporter: a new facet for adaptive responses to antidepressants. Science.

[23] Launay JM, Mouillet-Richard S, Baudry A, Pietri M, Kellermann O (2011). Raphe-mediated signals control the hippocampal response to SRI antidepressants via miR-16. Transl. Psychiatry.

[24] Song M-F (2015). CSF miR-16 is decreased in major depression patients and its neutralization in rats induces depression-like behaviors via a serotonin transmitter system. J. Affect. Disord..

[25] Bai M (2012). Abnormal hippocampal BDNF and miR-16 expression is associated with depression-like behaviors induced by stress during early life. PLoS ONE.

[26] Ma K, Guo L, Xu A, Cui S, Wang J-H (2016). Molecular mechanism for stress-induced depression assessed by sequencing miRNA and mRNA in medial prefrontal cortex. PLoS ONE.

[27] Liu Y, Liu D, Xu J, Jiang H, Pan F (2017). Early adolescent stress-induced changes in prefrontal cortex miRNA-135a and hippocampal miRNA-16 in male rats. Dev. Psychobiol..

[28] Zurawek D (2016). Time-dependent miR-16 serum fluctuations together with reciprocal changes in the expression level of miR-16 in mesocortical circuit contribute to stress resilient phenotype in chronic mild stress – an animal model of depression. Eur. Neuropsychopharmacol..

[29] Buran İ, Etem EÖ, Tektemur A, Elyas H (2017). Treatment with TREK1 and TRPC3/6 ion channel inhibitors upregulates microRNA expression in a mouse model of chronic mild stress. Neurosci. Lett..

[30] Xu C (2017). The interaction of miR-34b/c polymorphisms and negative life events increases susceptibility to major depressive disorder in Han Chinese population. Neurosci. Lett..

[31] Sun N (2020). Impact of expression and genetic variation of microRNA-34b/c on cognitive dysfunction in patients with major depressive disorder. Neuropsychiatr. Dis. Treat..

[32] Wan Y (2015). Identification of differential microRNAs in cerebrospinal fluid and serum of patients with major depressive disorder. PLoS ONE.

[33] Sun N (2016). Preliminary comparison of plasma notch-associated microRNA-34b and −34c levels in drug naive, first episode depressed patients and healthy controls. J. Affect. Disord..

[34] Bavamian S (2015). Dysregulation of miR-34a links neuronal development to genetic risk factors for bipolar disorder. Mol. Psychiatry.

[35] Zhou R (2009). Evidence for selective microRNAs and their effectors as common long-term targets for the actions of mood stabilizers. Neuropsychopharmacology.

[36] Kuang W-H, Dong Z-Q, Tian L-T, Li J (2018). MicroRNA-451a, microRNA-34a-5p, and microRNA-221-3p as predictors of response to antidepressant treatment. Brazilian J. Med. Biol. Res..

[37] Haramati S (2011). microRNA as repressors of stress-induced anxiety: the case of amygdalar miR-34. J. Neurosci..

[38] Mollinari C (2015). miR-34a regulates cell proliferation, morphology and function of newborn neurons resulting in improved behavioural outcomes. Cell Death Dis..

[39] Andolina D (2016). Effects of lack of microRNA-34 on the neural circuitry underlying the stress response and anxiety. Neuropharmacology.

[40] Li C, Liu Y, Liu D, Jiang H, Pan F (2016). Dynamic alterations of miR-34c expression in the hypothalamus of male rats after early adolescent traumatic stress. Neural Plast..

[41] Zhu J (2017). miR-34b attenuates trauma-induced anxiety-like behavior by targeting CRHR1. Int. J. Mol. Med..

[42] Lo Iacono L (2020). MicroRNA-34a regulates the depression-like behavior in mice by modulating the expression of target genes in the dorsal raphè. Mol. Neurobiol..

[43] Andolina D (2020). MicroRNA-34 contributes to the stress-related behavior and affects 5-HT prefrontal/GABA amygdalar system through regulation of corticotropin-releasing factor receptor 1. Mol. Neurobiol..

[44] Issler O (2014). MicroRNA 135 is essential for chronic stress resiliency, antidepressant efficacy, and intact serotonergic activity. Neuron.

[45] Mannironi C (2018). miR-135a regulates synaptic transmission and anxiety-like behavior in amygdala. Mol. Neurobiol..

[46] Kanasty RL, Whitehead KA, Vegas AJ, Anderson DG (2012). Action and reaction: the biological response to siRNA and its delivery vehicles. Mol. Ther..

[47] Bartoszewski R, Sikorski AF (2019). Editorial focus: understanding off-target effects as the key to successful RNAi therapy. Cell. Mol. Biol. Lett..

[48] Holsboer F (2000). The corticosteroid receptor hypothesis of depression clinical evidence. Neuropsychopharmacology.

[49] Anacker C, Zunszain PA, Carvalho LA, Pariante CM (2011). The glucocorticoid receptor: pivot of depression and of antidepressant treatment?. Psychoneuroendocrinology.

[50] Mendell JT (2008). miRiad roles for the miR-17–92 cluster in development and disease. Cell.

[51] Marques FZ (2017). A polymorphism in the norepinephrine transporter gene is associated with affective and cardiovascular disease through a microRNA mechanism. Mol. Psychiatry.

[52] Panta A, Pandey S, Duncan IN, Duhamel S, Sohrabji F (2019). Mir363-3p attenuates post-stroke depressive-like behaviors in middle-aged female rats. Brain. Behav. Immun..

[53] Pfau ML (2019). Role of monocyte-derived microRNA106b∼25 in resilience to social stress. Biol. Psychiatry.

[54] Rudisch B, Nemeroff CB (2003). Epidemiology of comorbid coronary artery disease and depression. Biol. Psychiatry.

[55] Hodes GE, Kana V, Menard C, Merad M, Russo SJ (2015). Neuroimmune mechanisms of depression. Nat. Neurosci..

[56] Camkurt MA (2020). MicroRNA dysregulation in manic and euthymic patients with bipolar disorder. J. Affect. Disord..

[57] Smalheiser NR (2014). Expression of microRNAs and other small RNAs in prefrontal cortex in schizophrenia, bipolar disorder and depressed subjects. PLoS ONE.

[58] Jin J (2016). miR-17–92 cluster regulates adult hippocampal neurogenesis, anxiety, and depression. Cell Rep..

[59] Volk N (2016). Amygdalar microRNA-15a is essential for coping with chronic stress. Cell Rep..

[60] Zannas AS, Wiechmann T, Gassen NC, Binder EB (2016). Gene–stress–epigenetic regulation of FKBP5: clinical and translational implications. Neuropsychopharmacology.

[61] Park H, Poo MM (2013). Neurotrophin regulation of neural circuit development and function. Nat. Rev. Neurosci..

[62] Björkholm C, Monteggia LM (2016). BDNF – a key transducer of antidepressant effects. Neuropharmacology.

[63] Ledonne A, Mercuri NB (2019). On the modulatory roles of neuregulins/ErbB signaling on synaptic plasticity. Int. J. Mol. Sci..

[64] Mei L, Nave KA (2014). Neuregulin-ERBB signaling in the nervous system and neuropsychiatric diseases. Neuron.

[65] Mei L, Xiong W-C (2008). Neuregulin 1 in neural development, synaptic plasticity and schizophrenia. Nat. Rev. Neurosci..

[66] Wei YBin (2016). MicroRNA 101b is downregulated in the prefrontal cortex of a genetic model of depression and targets the glutamate transporter SLC1A1 (EAAT3) in vitro. Int. J. Neuropsychopharmacol..

[67] Zhao Y (2017). MicroRNA-101 in the ventrolateral orbital cortex (VLO) modulates depressive-like behaviors in rats and targets dual-specificity phosphatase 1 (DUSP1). Brain Res.

[68] Zhu Q-Y, Liu Q, Chen J-X, Lan K, Ge B-X (2010). MicroRNA-101 targets MAPK phosphatase-1 to regulate the activation of MAPKs in macrophages. J. Immunol..

[69] Duric V (2010). A negative regulator of MAP kinase causes depressive behavior. Nat. Med..

[70] Monteggia LM (2004). Essential role of brain-derived neurotrophic factor in adult hippocampal function. Proc. Natl Acad. Sci. USA.

[71] Minichiello L (2009). TrkB signalling pathways in LTP and learning. Nat. Rev. Neurosci..

[72] Saus E (2010). Genetic variants and abnormal processing of pre-miR-182, a circadian clock modulator, in major depression patients with late insomnia. Hum. Mol. Genet..

[73] Li Y (2016). miR-182 (microRNA-182) suppression in the hippocampus evokes antidepressant-like effects in rats. Prog. Neuro-Psychopharmacol. Biol. Psychiatry.

[74] Zucchi FCR (2013). Maternal stress induces epigenetic signatures of psychiatric and neurological diseases in the offspring. PLoS ONE.

[75] Sheinerman KS, Tsivinsky VG, Abdullah L, Crawford F, Umansky SR (2013). Plasma microRNA biomarkers for detection of mild cognitive impairment: biomarker validation study. Aging (Albany NY).

[76] Sheinerman KS, Tsivinsky VG, Abdullah L, Crawford F, Umansky SR (2013). Plasma microRNA biomarkers for detection of mild cognitive impairment. Aging (Albany NY).

[77] Fiori, L. M. et al. miR-323a regulates ERBB4 and is involved in depression. *Mol. Psychiatry* (2020) 10.1038/s41380-020-00953-7. 10.1038/s41380-020-00953-7 33219358

[78] Wang W (2020). The protective role of neuregulin1-ErbB4 signaling in a chronic social defeat stress model. Neuroreport.

[79] Fan C (2018). miR-134 modulates chronic stress-induced structural plasticity and depression-like behaviors via downregulation of Limk1/cofilin signaling in rats. Neuropharmacology.

[80] Bamburg JR (1999). Proteins of the ADF/cofilin family: essential regulators of actin dynamics. Annu. Rev. Cell Dev. Biol..

[81] Schratt GM (2006). A brain-specific microRNA regulates dendritic spine development. Nature.

[82] Shen J (2019). The enriched environment ameliorates chronic unpredictable mild stress-induced depressive-like behaviors and cognitive impairment by activating the SIRT1/miR-134 signaling pathway in hippocampus. J. Affect. Disord..

[83] Zhang H (2020). Circulating microRNA 134 sheds light on the diagnosis of major depressive disorder. Transl. Psychiatry.

[84] Vo N (2005). From the cover: a cAMP-response element binding protein-induced microRNA regulates neuronal morphogenesis. Proc. Natl Acad. Sci. USA.

[85] Wayman GA (2008). An activity-regulated microRNA controls dendritic plasticity by down-regulating p250GAP. Proc. Natl Acad. Sci. USA.

[86] Impey S (2010). An activity-induced microRNA controls dendritic spine formation by regulating Rac1-PAK signaling. Mol. Cell. Neurosci..

[87] Edbauer D (2010). Regulation of synaptic structure and function by FMRP-associated microRNAs miR-125b and miR-132. Neuron.

[88] Hansen KF, Sakamoto K, Wayman GA, Impey S, Obrietan K (2010). Transgenic miR132 alters neuronal spine density and impairs novel object recognition memory. PLoS ONE.

[89] Mellios N (2011). miR-132, an experience-dependent microRNA, is essential for visual cortex plasticity. Nat. Neurosci..

[90] Tognini P, Putignano E, Coatti A, Pizzorusso T (2011). Experience-dependent expression of miR-132 regulates ocular dominance plasticity. Nat. Neurosci..

[91] Remenyi J (2010). Regulation of the miR-212/132 locus by MSK1 and CREB in response to neurotrophins. Biochem. J..

[92] Li YJ (2013). Alterations of serum levels of BDNF-related miRNAs in patients with depression. PLoS ONE.

[93] Su M, Hong J, Zhao Y, Liu S, Xue X (2015). MeCP2 controls hippocampal brain-derived neurotrophic factor expression via homeostatic interactions with microRNA-132 in rats with depression. Mol. Med. Rep..

[94] Liu Y (2016). Increased miR-132 level is associated with visual memory dysfunction in patients with depression. Neuropsychiatr. Dis. Treat..

[95] Qi S (2018). MicroRNA132 associated multimodal neuroimaging patterns in unmedicated major depressive disorder. Brain.

[96] Smalheiser NR (2011). MicroRNA expression in rat brain exposed to repeated inescapable shock: differential alterations in learned helplessness vs. non-learned helplessness. Int. J. Neuropsychopharmacol..

[97] Lin CC, Tsai MC, Lee C, Te, Sun MH, Huang TL (2018). Antidepressant treatment increased serum miR-183 and miR-212 levels in patients with major depressive disorder. Psychiatry Res..

[98] Chen H, Wang N, Burmeister M, McInnis MG (2009). MicroRNA expression changes in lymphoblastoid cell lines in response to lithium treatment. Int. J. Neuropsychopharmacol..

[99] Bocchio-Chiavetto L (2013). Blood microRNA changes in depressed patients during antidepressant treatment. Eur. Neuropsychopharmacol..

[100] Fang Y (2018). Changes in miRNA-132 and miR-124 levels in non-treated and citalopram-treated patients with depression. J. Affect. Disord..

[101] Ryan KM, O’Donovan SM, McLoughlin DM (2013). Electroconvulsive stimulation alters levels of BDNF-associated microRNAs. Neurosci. Lett..

[102] Hansen KF (2013). miRNA-132: a dynamic regulator of cognitive capacity. Brain Struct. Funct..

[103] Shaltiel G (2013). Hippocampal microRNA-132 mediates stress-inducible cognitive deficits through its acetylcholinesterase target. Brain Struct. Funct..

[104] Hansen KF (2016). Targeted deletion of miR-132/−212 impairs memory and alters the hippocampal transcriptome. Learn. Mem..

[105] Aten S (2019). miR-132/212 is induced by stress and its dysregulation triggers anxiety-related behavior. Neuropharmacology.

[106] Torres-Berrío A (2017). DCC confers susceptibility to depression-like behaviors in humans and mice and is regulated by miR-218. Biol. Psychiatry.

[107] Torres-Berrío A (2020). miR-218: a molecular switch and potential biomarker of susceptibility to stress. Mol. Psychiatry.

[108] Rocchi A (2019). Neurite-enriched microRNA-218 stimulates translation of the GluA2 subunit and increases excitatory synaptic strength. Mol. Neurobiol..

[109] Manitt C (2013). dcc Orchestrates the development of the prefrontal cortex during adolescence and is altered in psychiatric patients. Transl. Psychiatry.

[110] Inestrosa NC, Arenas E (2010). Emerging roles of Wnts in the adult nervous system. Nat. Rev. Neurosci..

[111] Chen G, Huang LD, Jiang YM, Manji HK (2000). The mood-stabilizing agent valproate inhibits the activity of glycogen synthase kinase-3. J. Neurochem..

[112] Okamoto H (2010). Wnt2 expression and signaling is increased by different classes of antidepressant treatments. Biol. Psychiatry.

[113] Makeyev EV, Zhang J, Carrasco MA, Maniatis T (2007). The microRNA miR-124 promotes neuronal differentiation by triggering brain-specific alternative pre-mRNA splicing. Mol. Cell.

[114] Cheng L-C, Pastrana E, Tavazoie M, Doetsch F (2009). miR-124 regulates adult neurogenesis in the subventricular zone stem cell niche. Nat. Neurosci..

[115] Uchida S (2010). Early life stress enhances behavioral vulnerability to stress through the activation of REST4-mediated gene transcription in the medial prefrontal cortex of rodents. J. Neurosci..

[116] Xu J (2017). FKBP5 and specific microRNAs via glucocorticoid receptor in the basolateral amygdala involved in the susceptibility to depressive disorder in early adolescent stressed rats. J. Psychiatr. Res..

[117] Xu J (2019). Short- and long-term alterations of FKBP5-GR and specific microRNAs in the prefrontal cortex and hippocampus of male rats induced by adolescent stress contribute to depression susceptibility. Psychoneuroendocrinology.

[118] Bahi A, Chandrasekar V, Dreyer JL (2014). Selective lentiviral-mediated suppression of microRNA124a in the hippocampus evokes antidepressants-like effects in rats. Psychoneuroendocrinology.

[119] Gu Z, Pan J, Chen L (2019). miR-124 suppression in the prefrontal cortex reduces depression-like behavior in mice. Biosci. Rep..

[120] Wang SS (2017). microRNA-124 targets glucocorticoid receptor and is involved in depression-like behaviors. Prog. Neuro-Psychopharmacol. Biol. Psychiatry.

[121] He S (2016). Alterations of microRNA-124 expression in peripheral blood mononuclear cells in pre- and post-treatment patients with major depressive disorder. J. Psychiatr. Res..

[122] Roy B, Dunbar M, Shelton RC, Dwivedi Y (2017). Identification of microRNA-124-3p as a putative epigenetic signature of major depressive disorder. Neuropsychopharmacology.

[123] Higuchi F (2016). Hippocampal microRNA-124 enhances chronic stress resilience in mice. J. Neurosci..

[124] Lou D, Wang J, Wang X (2019). miR-124 ameliorates depressive-like behavior by targeting STAT3 to regulate microglial activation. Mol. Cell. Probes.

[125] Deng ZF (2019). miR-214-3p targets β-catenin to regulate depressive-like behaviors induced by chronic social defeat stress in mice. Cereb. Cortex.

[126] Dias C (2014). β-catenin mediates stress resilience through dicer1/microRNA regulation. Nature.

[127] Enatescu VR (2016). Circulating plasma micro RNAs in patients with major depressive disorder treated with antidepressants: a pilot study. Psychiatry Investig..

[128] Lian N (2018). miR-221 is involved in depression by regulating Wnt2/CREB/BDNF axis in hippocampal neurons. Cell Cycle.

[129] Oved K (2013). Genome-wide expression profiling of human lymphoblastoid cell lines implicates integrin beta-3 in the mode of action of antidepressants. Transl. Psychiatry.

[130] Oved K (2017). MicroRNA-mediated regulation of ITGB3 and CHL1 is implicated in SSRI action. Front. Mol. Neurosci..

[131] Zhang Y (2020). CircDYM ameliorates depressive-like behavior by targeting miR-9 to regulate microglial activation via HSP90 ubiquitination. Mol. Psychiatry.

[132] Tavakolizadeh J (2018). MicroRNAs and exosomes in depression: potential diagnostic biomarkers. J. Cell. Biochem..

[133] Du Y (2019). Genome-wide, integrative analysis implicates exosome-derived microRNA dysregulation in schizophrenia. Schizophr. Bull..

[134] Wei ZX (2020). Exosomes from patients with major depression cause depressive-like behaviors in mice with involvement of miR-139-5p-regulated neurogenesis. Neuropsychopharmacology.

[135] Amoah SK (2020). Exosomal secretion of a psychosis-altered miRNA that regulates glutamate receptor expression is affected by antipsychotics. Neuropsychopharmacology.

[136] Bartel DP (2018). Metazoan microRNAs. Cell.

[137] Issler O, Chen A (2015). Determining the role of microRNAs in psychiatric disorders. Nat. Rev. Neurosci..

[138] Connelly CM, Uprety R, Hemphill J, Deiters A (2012). Spatiotemporal control of microRNA function using light-activated antagomirs. Mol. Biosyst..

[139] Himmerich H, Minkwitz J, Kirkby K (2015). Weight gain and metabolic changes during treatment with antipsychotics and antidepressants. Endocr., Metab. Immune Disord. Targets.

[140] Tardieu S, Micallef J, Gentile S, Blin O (2003). Weight gain profiles of new anti-psychotics: public health consequences. Obes. Rev..

[141] Beyer DKE, Freund N (2017). Animal models for bipolar disorder: from bedside to the cage. Int. J. Bipolar Disord..

[142] Czéh B, Fuchs E, Wiborg O, Simon M (2016). Animal models of major depression and their clinical implications. Prog. Neuro-Psychopharmacol. Biol. Psychiatry.

[143] Harro J (2019). Animal models of depression: pros and cons. Cell Tissue Res..

[144] Wang Q, Timberlake MA, Prall K, Dwivedi Y (2017). The recent progress in animal models of depression. Prog. Neuro-Psychopharmacol. Biol. Psychiatry.

[145] LeGates TA, Kvarta MD, Thompson SM (2019). Sex differences in antidepressant efficacy. Neuropsychopharmacology.

[146] Rubinow DR, Schmidt PJ (2019). Sex differences and the neurobiology of affective disorders. Neuropsychopharmacology.

[147] Simchovitz-Gesher A, Soreq H (2020). Pharmaceutical implications of sex-related RNA divergence in psychiatric disorders. Trends Pharmacol. Sci..

[148] Matos MR, Ho SM, Schrode N, Brennand KJ (2020). Integration of CRISPR-engineering and hiPSC-based models of psychiatric genomics. Mol. Cell. Neurosci..

[149] Viho EMG (2019). Corticosteroid action in the brain: the potential of selective receptor modulation. Neuroendocrinology.

[150] Torres-Berrío A, Hernandez G, Nestler EJ, Flores C (2020). The netrin-1/DCC guidance cue pathway as a molecular target in depression: translational evidence. Biol. Psychiatry.

